# Religious service attendance and mortality among older Black men

**DOI:** 10.1371/journal.pone.0273806

**Published:** 2022-09-02

**Authors:** Marino A. Bruce, Bettina M. Beech, Dulcie Kermah, Shanelle Bailey, Nicole Phillips, Harlan P. Jones, Janice V. Bowie, Elizabeth Heitman, Keith C. Norris, Keith E. Whitfield, Roland J. Thorpe

**Affiliations:** 1 Faith, Justice and Health and Men’s Health Collaboratories, University of Houston Population Health, University of Houston, Houston, TX, United States of America; 2 Department of Behavioral and Social Science, University of Houston Tilman J. Fertitta Family College of Medicine, Houston, TX, United States of America; 3 Department of Health Systems and Population Health Sciences, University of Houston Tilman J. Fertitta Family College of Medicine, Houston, TX, United States of America; 4 Program for Research on Men’s Health, Hopkins Center for Health Disparities Solutions, Johns Hopkins Bloomberg School of Public Health, Baltimore, MD, United States of America; 5 Charles R. Drew University School of Medicine and Science, Los Angeles, CA, United States of America; 6 Department of Microbiology, Immunology and Genetics, University of North Texas Health Science Center, Fort Worth, TX, United States of America; 7 Department of Health, Behavior and Society, Johns Hopkins Bloomberg School of Public Health, Baltimore, MD, United States of America; 8 Program Ethics in Science and Medicine, University of Texas Southwestern Medical Center, Dallas, TX, United States of America; 9 David Geffen School of Medicine at UCLA, Los Angeles, CA, United States of America; 10 University of Nevada-Las Vegas, Las Vegas, Nevada, United States of America; Yokohama City University, JAPAN

## Abstract

Religious institutions have been responsive to the needs of Black men and other marginalized populations. Religious service attendance is a common practice that has been associated with stress management and extended longevity. The objective of this study was to examine the relationship between religious service attendance and all-cause mortality among Black men 50 years of age and older. Data for this study were from NHANES III (1988–1994). The analytic sample (n = 839) was restricted to participants at least 50 years of age at the time of interview who self-identified as Black and male. Mortality was the primary outcome for this study and the NHANES III Linked Mortality File was used to estimate race-specific, non-injury-related death rates using a probabilistic matching algorithm, linked to the National Death Index through December 31, 2015, providing up to 27 years follow-up. The primary independent variable was religious service attendance, a categorical variable indicating that participants attended religious services at least weekly, three or fewer times per month, or not at all. The mean age of participants was 63.6±0.3 years and 36.4% of sample members reported that they attended religious services one or more times per week, exceeding those attending three or fewer times per month (31.7%), or not at all (31.9%). Cox proportional hazard logistic regression models were estimated to determine the association between religious service attendance and mortality. Participants with the most frequent religious service attendance had a 47% reduction of all-cause mortality risk compared their peer who did not attend religious services at all (HR 0.53, CI 0.35–0.79) in the fully adjusted model including socioeconomic status, non-cardiovascular medical conditions, health behaviors, social support and allostatic load. Our findings underscore the potential salience of religiosity and spirituality for health in Black men, an understudied group where elevated risk factors are often present.

## Introduction

Despite unparalled discovery in biomedical science and advances in clinical medicine, Black men in the United States continue to be disproportionately burdened by the early onset and accelerated progression of chronic diseases as well as disproportionately high levels of illness and complications from a wide array of health conditions [1–10]. The health and social challenges Black men perpetually face contribute to the truncated lifespan of this population. The poor health profile among this population have been noted in public discourse; however, a remarkably small number of investigators have explicitly examined morbidity and mortality among Black men [1,4,6,11]. The poor health and premature death of Black men is expensive as medical care expenditures and lost productivity cost have been estimated to be nearly $200 billion dollars [12].

Early work examining patterns of poor health and premature mortality among Black men focused primarily on maladaptive, health-compromising behavioral risk factors and social conditions (e.g., violence, unprotected sex, drug misuse). Although important, behavior is only one set of factors potentially contributing to disproportionate levels of premature mortality among this population. A growing number of health scientists have also begun to consider sociologic and psychologic factors potentially contributing to the early onset of disease and death among Black men. Recent studies have implicated stress as a major contributor to Black men’s health profiles [3,8,10,13–15]. Black men have been oppressed, commodified, surveilled, and criminalized like no other group in US history and they often experience disproportionately high levels of social and psychological stress from structural racism, institutional discrimination, and unfair treatment from early childhood through late adulthood [9,10,16]. The rapid accumulation and persistence of these stressors throughout the lifecourse are thought to be associated with increased mortality among Black men beginning as early as midlife [17,18]. Data from recent studies among the general population indicate that chronic stress contributes to activation of inflammatory processes, among others, leading to the onset and progression of chronic diseases; however, this and other pathways to disease development, disability, and premature mortality have not been well-explored, especially in Black men [9].

Current health promotion and disease prevention strategies incorporate stress management into protocols; however, few studies have examined how Black men cope with stress or assess how their coping strategies have implications for health outcomes [2,9]. A recent qualitative study of 150 Black men found that prayer or reading passages from sacred books were often used to cope with stressful circumstances [2]. Such practices, often referred to as *religiosity*, tend to be associated with social, doctrinal, and denominational characteristics of an organized religion [19,20]. Religious institutions under Black leadership are organizations in which Black men have been welcomed and serve as safe places for them to receive spiritual, social, emotional, and economic resources to help them cope with hostile and stressful social environments [9,21]. Attending religious services is a common form of religiosity among individuals later in the life [22–24]. This pattern is particularly salient for Black men because religious service attendance is highest during middle and late life for this group [9]. It is also noteworthy that recent studies have found religious service attendance among older Black men to be considerably higher than their White peers [25,26]. Religious service attendance has been associated with reduced mortality among adults in the general population [27–31] yet, very few studies have explicitly examined the relations between religiosity and health among Black men during middle and late life [9,26]. The purpose of this study is to examine the association between religious service attendance and all-cause mortality for middle- and older-age Black men in a nationally representative sample of adults in the US. We hypothesized that increased attendance of religious services is associated with a lower mortality rate among Black men.

## Materials and methods

### Survey design and data collection

The sample of Black men for this study was drawn from the Third National Health and Nutrition Examination Survey (NHANES III), a nationally representative sample of civilian, non-institutionalized persons from 89 random locations across the United States [32]. Respondents were selected and recruited using a stratified, multistage probability sampling design and their data were collected in two stages. Physical measurements and blood samples for laboratory analyses were collected during a study visit at the NHANES mobile examination center [33]. Social and demographic data were collected during a household interview. Data in NHANES III were collected between 1988–1994 in two phases (1988–1991 and 1991–1994), and included long-term follow-up, allowing for a robust assessment of mortality. NHANES III data are publicly available and de-identified. Analyses using these data are not classified as human subjects research and not subject to IRB review.

**Fig 1:**
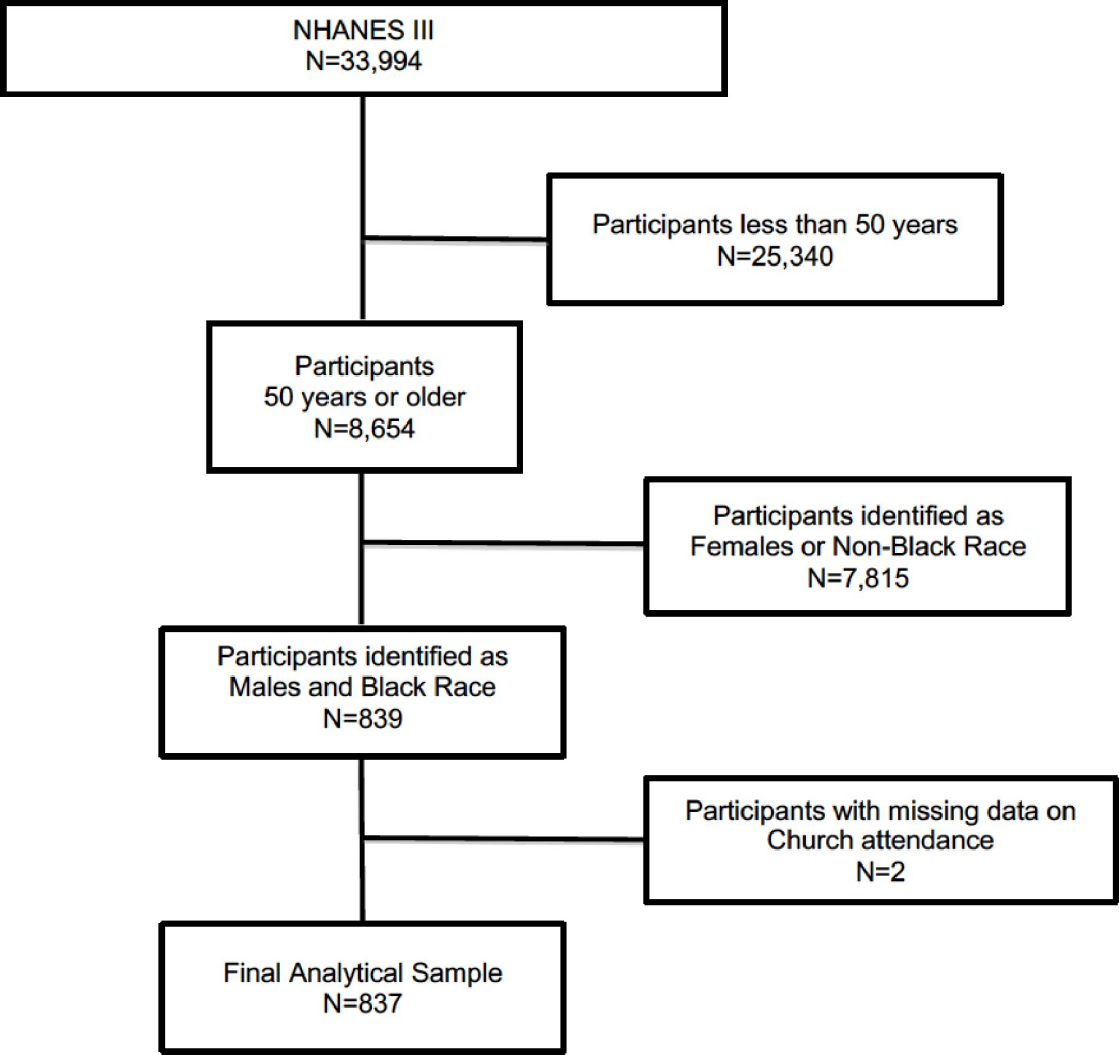
Algorithm used to define the study cohort.

Our study population was comprised of middle-age and older (at least 50 years of age at the time of interview [34]) males in NHANES III who self-identified as Black. Participants whose records were missing data on religious service attendance or two or more components of the allostatic load score (detailed below) were excluded. The analytic sample size for the study was 837 and the algorithm used to define the study cohort is depicted in Fig 1.

### Study variables

Mortality was the primary outcome for this study and the NHANES III Linked Mortality File was used to estimate race-specific, non-injury-related death rates for NHANES III participants using a probabilistic matching algorithm, linked to the National Death Index through December 31, 2015 [35] and provided up to 27 years follow-up (mean [SE]: 16 [0.2] years).

Religious service attendance was the primary independent variable of interest and was derived from an interview question asking respondents “How often do you attend church or religious services? (per year)”. The responses ranging from 0 to 1095 times a year were divided by 12 and classified into three categories, never attended, attended three or fewer times per month, and attended one or more times per week [27,28].

Allostatic load (AL) was calculated as a summative measure derived from values for 10 clinical/biologic variables available in NHANES III, which included cardiovascular measures (systolic blood pressure, diastolic blood pressure, total cholesterol/high density lipoprotein (HDL) ratio, homocysteine); nutritional/inflammatory markers (albumin, C-reactive protein); and metabolic (waist-hip ratio, glycated hemoglobin) biomarkers associated with physiologic dysregulation [36,37]. Dichotomous variables for each clinical/biological variable were derived to indicate high or low risk. Values above the 75^th^ percentile were defined as high risk and coded “1” for all biomarkers except HDL and albumin. High risk for these biomarkers were values below the 25^th^ percentile. The sum of the biomarker indicator variables with equal weighting was used to compute the total allostatic score with a range from 0–10 [38]. Age was a continuous variable derived from an item asking respondents to report their age in years.

Variables were also included to adjust for socioeconomic, behavioral, health, and social support factors that have been shown to be related to mortality [27,36]. The socioeconomic variables were education, health insurance, and income. Education was categorized as three variables denoting whether an individual completed <9, 9–12, or >12 years of education. Health insurance was coded as a dichotomous variable indicating whether or not a participant was insured. Income was measured by the poverty-income ratio [39], an income-to-needs variable measuring the ratio of household income to the US poverty threshold based on each respondent’s family size and composition at the time of the NHANES III examination [27,36]. Physical activity (any vs. none), smoking status, (current, former, never), alcohol use (non-drinker, 1–30 drinks/month, >30 drinks per month), and the Healthy Eating Index (HEI) [36] were behavioral variables. The health variables were self-report measures indicating whether respondents were told by a clinician that they had asthma, chronic obstructive pulmonary disease, non-skin cancer, thyroid disease, and rheumatoid arthritis as well as self-rated health. Self-rated health was assessed with a single item, “How would you rate your overall health?” with categories ranging from excellent to poor [30,40]. In this study self-rated health was categorized into three groups: “Excellent or Very Good”, “Good”, and “Fair or Poor” [30]. Social support measures for this study were drawn from questionnaire items asking respondents: a) “In a typical week, how many times do you talk on the telephone with family, friends, or neighbors?”, b) “How often do you get together with friends or relatives; I mean things like going out together or visiting in each other’s homes? (per year)”, c) “About how often do you visit with any of your other neighbors, either in their homes or in your own? (per year)” [27]. The natural logarithmic transformation of each social support variable was used in our analyses to compensate for skewness.

### Statistical analyses

Study population characteristics were described for the total sample and by church attendance, using means and standard errors for continuous variables and proportions for categorical variables. Cox proportional hazards analyses were used to generate hazard ratios (HR) and 95% confidence intervals (CI) for the total sample to examine the association between the level of church attendance and all-cause mortality among Black men. Model 1 included adjustment for race, age, sex, and non cardiovascular-related chronic conditions (asthma, chronic obstructive pulmonary disease, non-skin cancer, thyroid disease, rheumatoid arthritis), Model 2 added SES (education, poverty-income ratio, and health insurance status), to Model 1. Model 3 added health behaviors (smoking status, alcohol use, physical activity, and HEI) to Model 2. Model 4 added allostatic load score to Model 3, and Model 5 added self-rated health and social support indicators to Model 4. Adjusted survival curves for each attendance category were plotted using the Kaplan-Meier estimator and compared with the log-rank test.

NHANES III has a complex sampling design and all estimates were weighted to adjust for the differential probabilities of sampling and non-response, to represent the total civilian, non-institutionalized US population [41]. A p-value <0.05 was considered statistically significant. Final analyses were performed using SAS software V.9.4 (SAS Institute, Cary, North Carolina, USA), SUDAAN software Release 11.0.3 (SUDAAN Statistical Software Center, Research Triangle Park, North Carolina, USA), and STATA 16 (StataCorp, College Station, Texas).

## Results

The baseline characteristics of men in the study are provided in Table 1. All study participants were at least 50 years of age, with a mean cohort age of 63.6 ± 0.3 years. Over one-third of study participants (36.4%) reported that they attended religious services one or more times per week exceeding that of the other two attendance categories [3 or fewer times per month (31.7%), no attendance (31.9%)]. Black men with the most frequent religious service attendance were socioeconomically distinct from those who attended less frequently or not all. The proportion of respondents who reported attending religious services at least once per week was considerably greater than the other attendance categories among those reporting some college experience (23.7% vs 12.7% or 10.2%). Among respondents classified as poor, the proportion of individuals who attended religious service attendance one or times per week (49.5%) was considerably smaller than the segment of sample members in the other attendance categories (60.0%, 65.4%). The results in Table 1 indicate that Black men in the study who attended religious services at least once per week had a better overall health behavior profile than those who did not attend religious services. The proportion of never smokers (27.7%) and non-drinkers (63.1%) was highest among Black men who attended religious services at least once per week. The attendees of religious services at least once per week had the largest segment of individuals being physically active (67.0% vs 65.2% or 47.0%) and the highest mean Healthy Eating Index scores (61.5 ± 0.8 vs 57.5 ± 0.8 or 57.0 ± 0.9). Only one of the social support variables had notable association with religious service attendance: Black men in the study who attended religious services at least once per week averaged fewer weekly phone calls with family, friends, and (7.0 ± 1.0) than those who attended 3 or fewer times per month (10.0 ± 1.0) or not at all (14.0 ± 1.0).

**Table 1:** Baseline characteristics of Black Men 50 and older in NHANES III by self-reported religious service attendance.

	Total Sample (n = 837)	1+ times per week (n = 305)	≤ 3 times per month (n = 265)	No attendance (n = 267)	*P*
Mean age (yrs) [Mean (SE)]	63.6 (0.3)	63.9 (0.7)	63.3 (0.6)	63.7 (0.7)	0.854
Mean allostatic load score [Mean (SE)]	3.2 (0.1)	3.2 (0.1)	3.3 (0.1)	3.1 (0.1)	0.74
Education [n,%]					*<0*.*001*
<High school	363 (38.1)	112 (31.1)	123 (40.6)	127 (43.8)	
High school/GED	353 (45.9)	136 (45.2)	107 (46.7)	110 (46.0)	
Some college +	109 (16.0)	56 (23.7)	28 (12.7)	25 (10.2)	
Poor (poverty-income ratio<2) [n,%]	467 (58.0)	146 (49.5)	152 (60.0)	168 (65.4)	*0*.*001*
No health insurance [n,%]	45 (6.3)	16 (5.4)	13 (6.5)	16 (7.0)	*0*.*719*
Self-rated health [n,%]					*0*.*007*
Excellent/Very good	209 (26.3)	81 (28.7)	70 (27.7)	58 (22.7)	
Good	291 (35.6)	123 (41.3)	81 (31.0)	86 (33.2)	
Fair/Poor	339 (38.1)	101 (30.1)	114 (41.3)	123 (44.1)	
Smoking [n,%]					*<0*.*001*
Never smoker	209 (25.2)	84 (27.7)	75 (30.7)	50 (17.4)	
Former smoker	344 (39.4)	156 (50.1)	94 (31.8)	94 (34.6)	
Current smoker	285 (35.4)	65 (22.2)	96 (37.5)	123 (48.0)	
Physically active [n,%]	485 (59.8)	201 (67.0)	168 (65.2)	116 (47.0)	*<0*.*001*
Alcohol use [n,%]					*<0*.*001*
Non-drinkers	463 (51.5)	203 (63.1)	135 (46.4)	125 (43.5)	
1–30 drinks/month	303 (39.5)	98 (35.5)	103 (43.0)	101 (40.6)	
>30 drinks/month	71 (9.0)	4 (1.4)	27 (10.6)	39 (15.9)	
HEI score [Mean (SE)]	58.8 (0.5)	61.6(0.8)	57.5(0.8)	57.0(0.9)	*<0*.*001*
Non CV comorbidities [n,%]					
Lung disease	70 (7.6)	22 (6.2)	16 (5.4)	31 (10.8)	*0*.*016*
Cancer	44 (4.9)	15 (5.1)	15 (5.2)	14 (4.6)	*0*.*942*
Thyroid disease	17 (1.8)	7 (2.2)	5 (1.4)	5 (1.7)	*0*.*721*
Rheumatoid arthritis	52 (6.0)	18 (5.7)	14 (4.8)	20 (7.7)	*0*.*322*
Asthma	51 (6.9)	14 (6.9)	17 (6.4)	20 (7.5)	*0*.*938*
Social support [Mean (SE)]					
Number of phone calls with family, friends, or neighbors/week	9 (1.0)	7 (1)	10 (1)	14 (1)	*0*.*01*
Number of visits with friends or relatives/year	69 (1.0)	71 (1)	71 (1)	64 (1)	*0*.*6*
Number of visits with other neighbors/year	83 (1.0)	71 (1)	97 (1)	88 (1)	*0*.*2*

^a^Estimate is unreliable, as the sample size was smaller than that recommended in the NHANES analytic guidelines for the design effect and estimated proportion [22,23].

The data presented are the weighted percentages, which may not add up to 100.

SE: Standard error; CV-cardiovascular; HEI—Healthy Eating Index; GED- General Educational Diploma; NHANES III- Third National Health and Nutrition Examination Survey.

**Fig 2:**
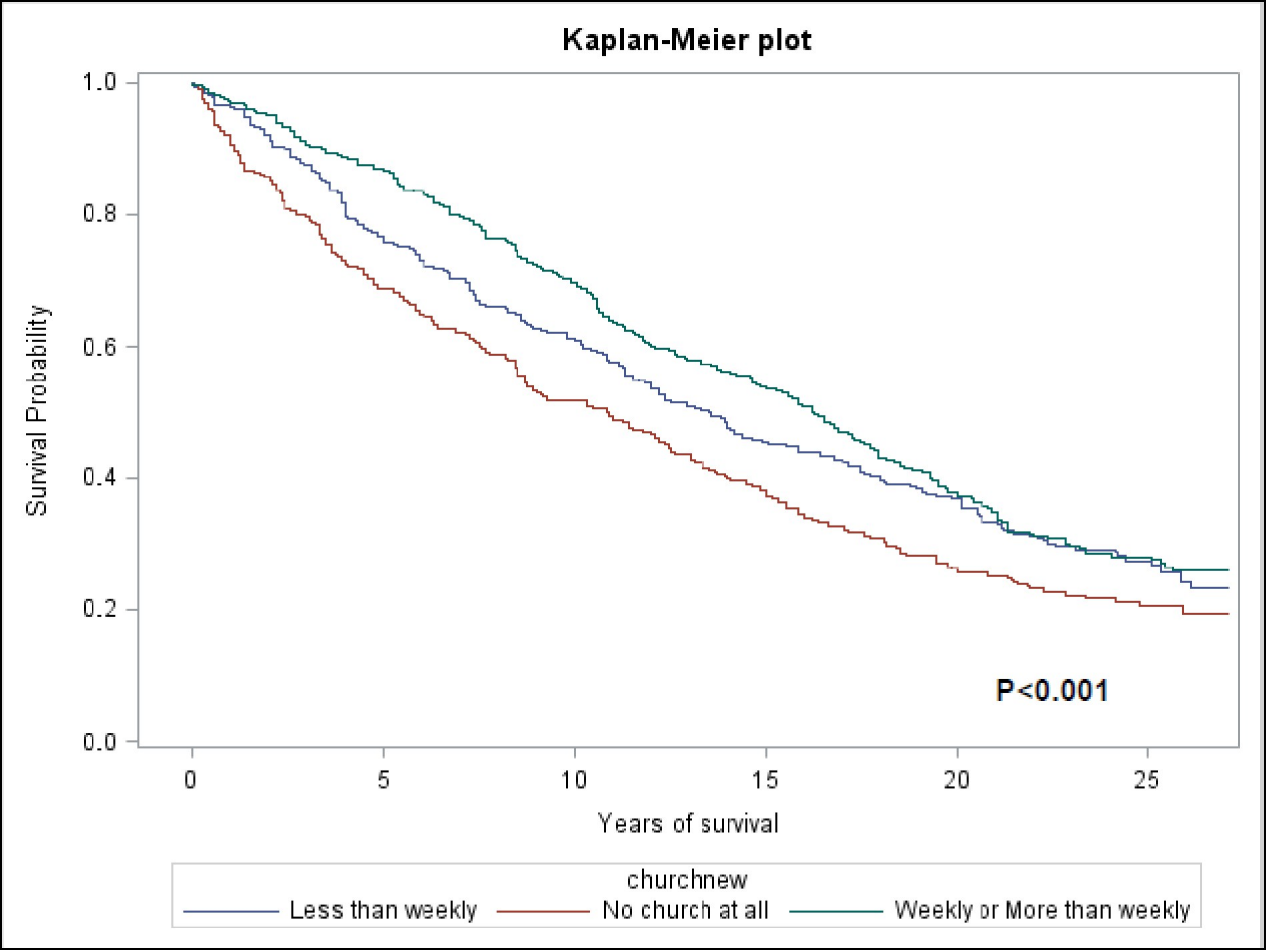
Unadjusted Kaplan-Meier curves for all-cause mortality by church attendance.

The association between religious service attendance and all-cause mortality among Black men is displayed in Fig 2 and Table 2. The unadjusted Kaplan-Meier curves presented in Fig 2 indicate that survival was highest among Black men who attended religious services at least weekly and lowest among those who did not attend at all. Black men who attended religious services at least one per week had a lower risk for all-cause mortality than sample members who did not attend religious services across all of the hazard models presented in Table 2. Black men who attended religious services at least once per week had a highly statistically significant HR of 0.53 (95% CI: 0.35–0.79) for all-cause mortality relative to those who did not attend religious services, after adjusting for health, socioeconomic, behavioral, allostatic load, and social support.

**Table 2:** Hazard ratios for all-cause mortality by religious service attendance for Black Men 50 and older in NHANES III.

	Unadjusted	Adjusted			
		Model 1	Model 2	Model 3	Model 4
No attendance	Reference	Reference	Reference	Reference	Reference
**Attendance 3 or fewer times/month**	0.79 (0.64–0.96)	0.76 (0.57–1.02)	0.83 (0.61–1.11)	0.70 (0.46–1.07)	0.70 (0.46–1.07)
**Attendance one or more times/week**	0.71 (0.58–0.86)	0.59 (0.44–0.77)	0.62 (0.45–0.85)	0.51 (0.33–0.78)	0.53 (0.35–0.79)

NHANES III—Third National Health and Nutrition Examination Survey.

• Model 1 adjusts for age, asthma, chronic obstructive pulmonary disease, non-skin cancer, thyroid disease, rheumatoid arthritis, social support, and self-rated health.

• Model 2 adds education, poverty-income ratio, and health insurance status to the covariates in Model 1.

• Model 3 adds health behaviors and the healthy eating index score to the covariates in Model 2.

• Model 4 adds allostatic load score to the covariates in Model 3.

## Discussion

Advances in science and healthcare have allowed individuals in the United States to live longer; however, the life expectancy for Black men remains significantly shorter than for other groups of women and men [42]. Religious service attendance has been associated with improvements in well-being and extended longevity, especially in later life [27]. Our study is the first study to examine association between religious service attendance and mortality risk in a sample of Black men in the United States who are at least 50 years of age. We found that Black men who attended religious services at least once a week had a lower mortality risk than their peers who did not attend religious services. This association was minimally impacted by adjustment for demographic, social, economic, behavioral, and clinical variables, including allostatic load. These findings emphasize the potential independent role of faith-related practices for extending life for Black men during middle and later life.

Our findings are consistent with other studies demonstrating religious service attendance to be associated with lower mortality risk in general populations [27,29,43]. This is important because our study is one of a few studies examining the degree to which religiosity is salutary for Black men’s health and longevity. Black religious institutions have been responsive to an array of individual and community needs, suggesting that the association between frequency of religious service attendance and mortality among middle-age and older Black men can be attributed to multiple factors. Black men tend to have an earlier onset of chronic diseases, more aggressive disease progression, and more complications than other groups of men. Chronic disease can present multiple stressors including burdensome therapeutic protocols and the loss of income, productivity, and quality of life [21,44]. It has been suggested that religious settings and services present opportunities for individuals to have spiritually enriching experiences and to receive emotional support and affirmative messages useful for coping with the uncertainty, vulnerability, hopelessness, fear, anger, and particularly depression that can accompany chronic conditions [21,26,45]. Religious institutions can also be sources of social and economic support to obtain important health information and services [9,46,47]. The impact of attending religious services on physiological processes and clinical outcomes among individuals with chronic health conditions is not clear; however, our results suggest that future studies are critically needed examining the influence that close association with a religious body can have on health and longevity of among marginalized populations with chronic conditions.

Black religious institutions can also be contexts for promoting resiliency and resistance against forces such as the structural racism that is often the primary source of discriminatory practices that adversely impact the lives of Black men. Black houses of worship have traditionally been in unique positions to advocate for Black communities and residents because they are one of few social institutions built, financed, and controlled by Black leadership [48–50]. This independence has allowed them to advocate for the communities they serve. Black religious institutions guided by theological perspectives in which liberation from oppression is a central tenet have been influential in collective actions large and small [51–53]. Regular engagement in communities with this orientation can buffer the impact of marginalization, foster a sense of connection with others [54,55], and instill meaning and purpose regardless of life circumstances [27,56]. Affirmative environments can also encourage personal or individual-level religious practices (e.g., prayer, meditation) that can reduce stress and mitigate maladaptive biological (e.g., elevated blood pressure) and behavioral (e.g., substance abuse) responses often associated with the onset, progression, and complications of chronic disease. Recent studies have presented evidence suggesting that prayer, meditation, and personal religious or spiritual practices can be effective for treating post-traumatic stress disorder [57], reduce cardiovascular disease risks [58–61], and potentially prolong longevity [27,62].

Our findings underscore the potential salience of religiosity and spirituality for health in Black men, a group that has been understudied and where elevated risk factors are often present. This study contributes to the existing body of minority health and men’s health research; however, it has some noteworthy limitations. Religious service attendance has been used as a proxy for religious practice; however, it is a single outward expression of religiosity. Our study does not account for private practices such as prayer or reading sacred texts nor the heterogeneity of religious services across and within denominations or sects [63]. We adjusted for known risk factors associated with mortality, including major medical conditions and allostatic load, but we could not account for residual confounding such as risk factor severity or duration, depression, religious affiliation or other unmeasured factors in the model. At the writing of this manuscript, the National Death Index match only goes through 2015. Data used for this study are observational and causality cannot be inferred from our findings. Our research includes self-report data, and limitations such as recall bias and social desirability apply.

Despite these limitations, our study demonstrates a powerful association with reduced mortality that, after adjusting for common mortality risk factors, supports the potential survival benefits of attending religious services for middle age and older Black men. Data from this research provides some intriguing avenues of inquiry at the interface of religiosity, spirituality, and health. Nuanced considerations of factors such as racism, gender, status in society, stress, religious participation, and religious coping lay the foundation for the next generation of studies investigating how religious participation and other forms of religiosity and spirituality can get “under the skin” to influence physiological and cognitive functioning as well as promote longevity among Black men and other populations that have been historically marginalized.
